# MicroRNAs Located in the Hox Gene Clusters Are Implicated in Huntington's Disease Pathogenesis

**DOI:** 10.1371/journal.pgen.1004188

**Published:** 2014-02-27

**Authors:** Andrew G. Hoss, Vinay K. Kartha, Xianjun Dong, Jeanne C. Latourelle, Alexandra Dumitriu, Tiffany C. Hadzi, Marcy E. MacDonald, James F. Gusella, Schahram Akbarian, Jiang-Fan Chen, Zhiping Weng, Richard H. Myers

**Affiliations:** 1 Department of Neurology, Boston University School of Medicine, Boston, Massachusetts, United States of America; 2 Graduate Program in Genetics and Genomics, Boston University School of Medicine, Boston, Massachusetts, United States of America; 3 Bioinformatics Program, Boston University, Boston, Massachusetts, United States of America; 4 Program in Bioinformatics and Integrative Biology, and Department of Biochemistry and Molecular Pharmacology, University of Massachusetts Medical School, Worcester, Massachusetts, United States of America; 5 Center for Human Genetic Research, Massachusetts General Hospital, Harvard Medical School, Boston, Massachusetts, United States of America; 6 Friedman Brain Institute, Department of Psychiatry, Mount Sinai School of Medicine, New York, New York, United States of America; 7 Genome Science Institute, Boston University School of Medicine, Boston, Massachusetts, United States of America; University of California, United States of America

## Abstract

Transcriptional dysregulation has long been recognized as central to the pathogenesis of Huntington's disease (HD). MicroRNAs (miRNAs) represent a major system of post-transcriptional regulation, by either preventing translational initiation or by targeting transcripts for storage or for degradation. Using next-generation miRNA sequencing in prefrontal cortex (Brodmann Area 9) of twelve HD and nine controls, we identified five miRNAs (miR-10b-5p, miR-196a-5p, miR-196b-5p, miR-615-3p and miR-1247-5p) up-regulated in HD at genome-wide significance (FDR q-value<0.05). Three of these, miR-196a-5p, miR-196b-5p and miR-615-3p, were expressed at near zero levels in control brains. Expression was verified for all five miRNAs using reverse transcription quantitative PCR and all but miR-1247-5p were replicated in an independent sample (8HD/8C). Ectopic miR-10b-5p expression in PC12 HTT-Q73 cells increased survival by MTT assay and cell viability staining suggesting increased expression may be a protective response. All of the miRNAs but miR-1247-5p are located in intergenic regions of Hox clusters. Total mRNA sequencing in the same samples identified fifteen of 55 genes within the Hox cluster gene regions as differentially expressed in HD, and the Hox genes immediately adjacent to the four Hox cluster miRNAs as up-regulated. Pathway analysis of mRNA targets of these miRNAs implicated functions for neuronal differentiation, neurite outgrowth, cell death and survival. In regression models among the HD brains, huntingtin CAG repeat size, onset age and age at death were independently found to be inversely related to miR-10b-5p levels. CAG repeat size and onset age were independently inversely related to miR-196a-5p, onset age was inversely related to miR-196b-5p and age at death was inversely related to miR-615-3p expression. These results suggest these Hox-related miRNAs may be involved in neuroprotective response in HD. Recently, miRNAs have shown promise as biomarkers for human diseases and given their relationship to disease expression, these miRNAs are biomarker candidates in HD.

## Author Summary

Huntington's disease (HD) is an inherited fatal neurological disorder that commonly affects people in midlife. Past studies have implicated abnormal patterns of gene expression as a candidate for causing the death of the brain cells affected in HD. MicroRNAs (miRNAs) are small molecules that regulate and target transcripts for either storage or destruction. We measured the levels of miRNAs, as well as the levels of gene expression (mRNAs) in twelve HD and nine control brain samples. We found five miRNAs that had greatly increased expression in the HD brains, including three that were not expressed in the normal samples. Four of these were related to important characteristics of the disease expression, including the age at disease onset, and the age at death of the individual. The genes that these miRNAs target for regulation were also altered in their expression with most being increased, suggesting they may have been targeted for storage. One of the miRNAs, miR-196a-5p was previously implicated in enhancing the survival of brain cells in HD. When we overexpressed miR-10b-5p in an HD cell model, the cells survived longer than untreated cells, suggesting these miRNAs may promote neuron survival and may hold new clues for treatments in HD.

## Introduction

Huntington's disease (HD) (OMIM: 143100) is an inherited neurodegenerative disorder characterized by involuntary movement, dementia, and changes in personality. HD is transmitted as an autosomal dominant disorder, for which an expansion of a CAG trinucleotide repeat within the coding region of the huntingtin gene (*HTT*) is the disease causing mutation [1]. The CAG repeat codes for a polyglutamine domain in the Htt protein and results in neuronal cell death predominantly affecting the caudate nucleus and putamen although neuronal loss is widespread in the HD brain [2], [3]. While the biological processes leading to neurodegeneration in HD are poorly understood, transcriptional dysregulation has long been proposed as central to the pathogenesis of HD. Widespread alterations in gene expression have been reported [4] and several studies suggest that gene expression may be altered at one or more of the stages of RNA processing, translation, protein post-translational modification or trafficking [5], [6].

MicroRNAs (miRNAs) are small non-coding RNAs that function as translational regulators of mRNA expression. miRNAs may inhibit gene expression either by repressing translation, or by targeting mRNA for either storage or degradation [7]. Recently, dysregulation of miRNAs has been linked to neurological and neurodegenerative disorders [8] and several studies have explored the role of miRNAs in HD. Marti et al [9] performed miRNA-sequencing for two pooled HD samples and two pooled control samples and reported altered expression for a large number of miRNAs. Altered expression of miRNAs, quantified using microarray technology, has been reported in cellular models of HD [10]–[12] and in mouse models of HD [12]–[15] but a comprehensive study of miRNA and mRNA expression obtained through next-generation sequencing technology in human HD samples has not been performed.

In order to investigate (1) the presence of altered miRNA expression and (2) the potential role of miRNAs on the altered mRNA expression seen in HD, we performed both miRNA-sequencing and mRNA sequencing, using Illumina massively parallel sequencing in twelve HD and nine neurologically normal control brains. To our knowledge this is the first genome-wide quantification of miRNA expression comparing human HD and control brain, and the first to combine total miRNA expression with total mRNA expression obtained through massively parallel sequencing.

## Results

### Selection of prefrontal cortex and BA9

While the striatum is the region most heavily involved neuropathologically in HD [3], 80% to 90% of the neurons in that region will have degenerated by the time of death. These changes, together with the presence of reactive astrocytosis, alter the cellular composition of the striatum. In contrast, cortical involvement in HD is well defined [2], [16] and while it does not experience dramatic neuronal degeneration, cortical neurons are known to exhibit the effects of protein aggregation and nuclear inclusion bodies characteristic of the disease. Therefore, we selected the prefrontal cortex for these studies.

### Five miRNAs are up-regulated in HD

**Figure 1:**
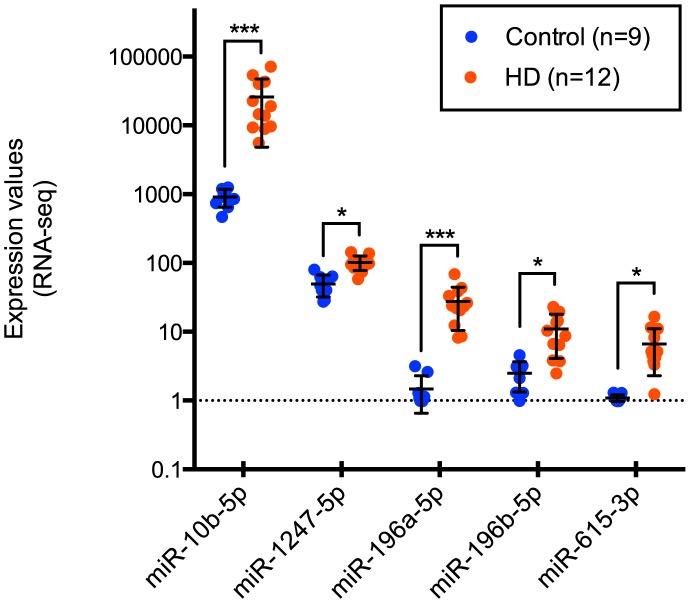
Differentially expressed miRNAs in Huntington's disease. miR-10b-5p, miR-1247-5p, miR-196a-5p, miR-196b-5p, and miR-615-3p were identified as differentially expressed in Huntington's disease prefrontal cortex compared to non-neurological disease controls by Illumina miRNA-sequencing. Normalized expression values quantified from DESeq analysis are shown on the y-axis. miR-196a-5p, miR-196b-5p and miR-615-3p were essentially not expressed in control samples, while the mean HD expression was 27.49, 11.01 and 6.66 respectively. miR-1247-5p was expressed at moderate levels in both control (mean = 49.44) and HD brain (mean = 102.01). miR-10b-5p was expressed in control (mean = 915.81) and highly expressed in HD brain (mean = 26,020.05). For miRNA, *p<0.05 and ***p<0.001, as determined by DESeq, followed by the Benjamini-Hochberg multiple comparison correction. (HD = Huntington's disease).

After removing sample outliers using principal component analysis filtering, we identified five out of 1,417 detected mature miRNA species as differentially expressed between twelve HD and nine control prefrontal cortex samples using the R statistical package DESeq (**Tables 1** **,** **2** **and** **3** **;** **Figure 1**). All five miRNAs were significantly up-regulated in HD. The largest effect between conditions was seen for miR-10b-5p, with a 28.41 fold increased expression in HD relative to control samples (mean control expression = 915.81; mean HD expression = 26,020.05, Figure 1). miR-1247-5p was expressed at moderate levels in both control (mean = 49.44) and HD brain (mean = 102.01). Three of the miRNAs, miR-196a-5p (mean control expression = 1.47; mean HD expression = 27.49), miR-196b-5p (mean control expression = 2.49; mean HD expression = 11.01) and miR-615-3p (mean control expression = 1.09, mean HD expression = 6.66), had near zero expression levels in all nine control samples.

**Table 1:** HD brain samples analyzed for mRNA-seq, miRNA-seq and RT-qPCR validation of miR-10b-5p.

Sample ID	miRNA-seq	RT-qPCR	PMI (hr.)	RIN or RQN	Death age	Onset age	Duration (yr.)	CAG repeat size	Neuron Loss in Neocortical Gray Matter
HD-01	Passed	Y	37	7.1	55	44	11	45	1
HD-02	Passed	Y	6	7.5	69	63	6	41	1
HD-03	Passed	Y	21	7	71	52	19	43	1
HD-05	Passed	Y	19	6.9	48	25	23	48	2
HD-06	Passed	Y	NA	6.2	40	34	6	51	1
HD-07	Passed	Y	8	8.5	72	55	17	41	1
HD-08	Passed	Y	21	7.4	43	NA	NA	49	1
HD-09	Passed	Y	4	7.8	68	45	23	42	1
HD-10	Passed	Y	6	8.3	59	35	24	46	1
HD-12	Passed	Y	13	6	68	52	16	42	0
HD-13	Passed	N	25	6.1	57	40	17	49	1
HD-14	Passed	Y	11	7.3	48	38	10	45	1
Mean	-	-	15.48	7.18	58.17	43.91	15.64	45.17	0.875

All of the HD samples passed mRNA-seq QC.

Scale of neuron loss: 0 = absent, 1 = mild, 2 = moderate.

**Table 2:** Control brain samples analyzed for mRNA-seq, miRNA-seq and RT-qPCR validation of miR-10b-5p.

Sample ID	miRNA-seq	RT-qPCR	PMI (hr.)	RIN or RQN	Death age
C-14	Passed	Y	21	8	79
C-21	Passed	Y	26	7.3	76
C-29	Passed	Y	13	6.4	93
C-31	Passed	Y	24	7.3	53
C-32	Passed	Y	24	8.3	57
C-33	Passed	Y	15	7.5	43
C-35	Failed PCA	N	21	7.6	46
C-36	Passed	Y	17	7.5	40
C-37	Failed PCA	N	28	8.3	44
C-38	Passed	Y	20	7.7	57
C-39	Passed	Y	15	7.3	80
Mean	-	-	20.36	7.49	60.73

All the control samples passed mRNA-seq QC.

RIN = RNA Integrity Number, RQN = RNA Quality Number.

PMI = Postmortem Interval.

**Table 3:** Differentially expressed miRNAs from miRNA-seq.

miRNA	Control expression	HD expression	Fold Change	p-value	q-value*
miR-196a-5p	1.47	27.49	18.66	2.05E-10	2.91E-07
miR-10b-5p	915.81	26020.05	28.41	1.99E-08	1.41E-05
miR-615-3p	1.09	6.66	6.09	2.73E-05	1.29E-02
miR-1247-5p	49.44	102.01	2.06	7.67E-05	2.72E-02
miR-196b-5p	2.49	11.01	4.41	9.77E-05	2.77E-02

* FDR-adjusted q-value.

### Validation and replication of miRNA findings

miRNA expression differences were orthogonally validated using the Exiqon miRCURY LNA technology for reverse transcription quantitative PCR (RT-qPCR) in eleven of twelve sequenced HD samples and nine control samples originally studied for miRNA-seq. All five miRNAs were confirmed to be significantly up-regulated in HD (**Table S1**), consistent with our miRNA-sequencing findings.

To replicate our findings in an independent sample set, we performed RT-qPCR in an additional eight control and eight HD prefrontal cortical samples (**Tables S2 and S3**). Four out of five miRNA (miR-10b-5p, miR-196a-5p, miR-196b-5p, miR-615-3p) were confirmed as significantly increased in expression in HD (**Table S4**).

### Similar proportion of neurons in HD and control cortical brain homogenate samples

HD is characterized by progressive cortical atrophy, with recognizable neuropathologic abnormalities in the neocortical gray matter [2], [16]–[20] (**Table 1**). To address whether miRNA expression changes in HD may be due to altered ratios in brain cell-type abundance, such as a change in the ratio of neurons to glial cells, we compared the number of neuronal and non-neuronal nuclei across conditions. Suspensions of cell nuclei of prefrontal cortex from 28 HD cases and 19 controls were immunocytochemically labeled with anti-NeuN, a neuron-specific nuclear antigen, followed by flow cytometric analysis. The mean and range of NeuN+ ratios for controls and cases were not significantly different (t = 1.67, p-value = 0.10; **Figure S1**), suggesting cortical neuron loss in the BA9 area in HD is relatively modest and does not account for the dramatic alterations in miRNA levels reported here.

### Increased miR-10b-5p expression is not observed in Parkinson's disease (PD)

To establish whether miR-10b-5p change is a generalized response to neurodegeneration, we evaluated this miRNA in PD prefrontal cortex. While cortical neuronal loss is variable in PD, both PD and HD are neurodegenerative and caused by protein inclusions. We selected PD prefrontal cortex samples that exhibited reported neuron loss on their neuropathological evaluation (n = 6) and PD samples without reported cortical neuronal loss (n = 8). From total RNA, RT-qPCR was performed for miR-10b-5p (**Table S5**). No difference was seen in miR-10b-5p expression when stratifying PD based on the extent of neuron loss (t = 0.59, p-value = 0.58). Additionally, no significant difference in HD miR-10b-5p expression from qPCR was observed when stratifying HD cases based on a measure of cortical neuron loss (f = 0.28, p-value = 0.76).

**Figure 2:**
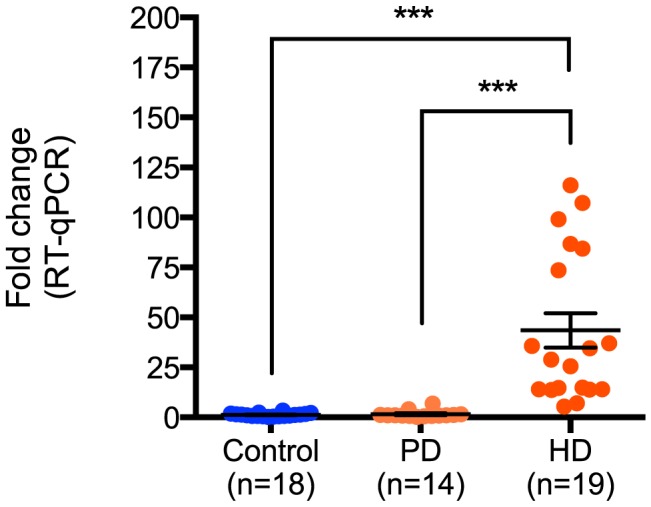
miR-10b-5p expression in control, Parkinson's disease and Huntington's disease prefrontal cortex. Up-regulation of miR-10b-5p was confirmed in HD by performing RT-qPCR, comparing nineteen Huntington's disease prefrontal cortex samples to eighteen non-neurological disease control samples (***p<0.001) or fourteen Parkinson's disease samples (***p<0.001). ΔΔC_T_ values of miR-10b-5p in PD and HD as compared to controls are shown on the y-axis. The absence of up-regulation in PD frontal cortex suggests that up-regulation of miR-10b-5p may be HD specific. (C_T_ = cycle threshold; RT-qPCR = reverse transcription quantitative PCR; PD = Parkinson's disease; HD = Huntington's disease).

Next, the relative expression of miR-10b-5p in PD was compared to all nineteen HD and eighteen control samples assayed. While no significant difference in miR-10b-5p expression was observed between control and PD samples (q = 0.05, p = 0.99), a significant difference was seen in HD compared to PD (q = 7.30, p<0.0001; **Figure 2**), suggesting increased miR-10b-5p expression, independent of neuron loss, is not a generalized response to neurodegeneration.

### Ectopic miR-10b-5p expression protects HD cell lines from polyglutamine-mediated cytotoxicity

To determine the functional importance of miR-10b-5p up-regulation in HD, we ectopically expressed miR-10b-5p in PC12 Q73 cells. These cell stably expressed huntingtin fragment derived from exon 1 (1–90), contain a pathogenic, 73 long polyglutamine repeat and a MYC epitope for protein identification. PC12 cells have been shown to terminally differentiate and form neural processes upon nerve growth factor (NGF) treatment [21], and HD models of these cells have been highly characterized, exhibiting phenotypic changes such as aggregate formation and polyglutamine-dependent cell death [22]–[26].

**Figure 3:**
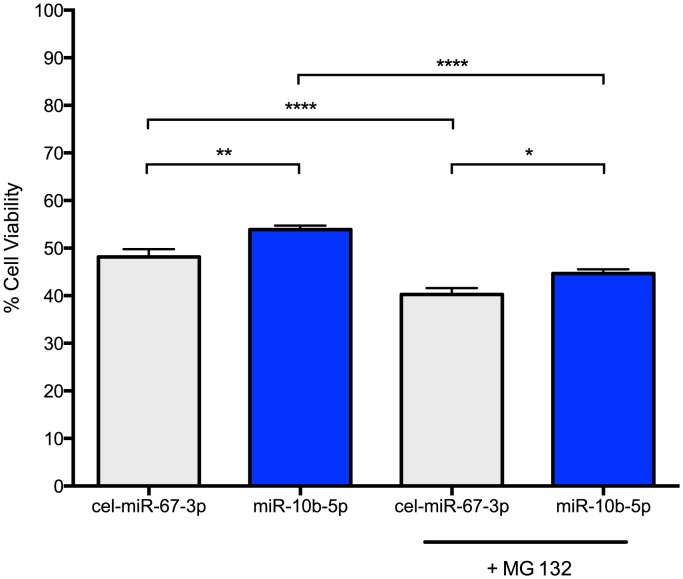
miR-10b-5p overexpressing PC12 Q73 cells exhibit reduced cytotoxicity. PC12 cells expressing huntingtin exon 1 with a polyglutamine expansion spanning 73 repeats were transfected with miR-10b-5p or cel-miR-67-3p as a negative control. On day 3 post-differentiation, a subset of cells were treated with 1 uM MG 132. A MTT assay was used to measure cell viability after four days post differentiation. On the Y-axis, the viability percentage was calculated from the initial cell count. Error bars represent SEM. (****p<0.0001; **p<0.001 *p<0.05).

PC12 Q73 cells were transfected with miR-10b-5p mimic or a negative control mimic, cel-miR-67-3p, after 48 hours post-differentiation. Cell survival was quantified using a MTT cell viability assay 48 hours post-transfection. Increased survival, though modest (53.9% versus 48.2%), was statistically higher for cells transfected with miR-10b-5p compared to cells transfected with negative control miRNA (q = 4.58, p-value<0.0001; **Figure 3**). The enhanced survival via ectopic miR-10b-5p expression was further substantiated in experiments using viable fluorescent cell staining, where miR-10b-5p transfected cells showed increased cell viability over cells transfected with negative control miRNA (t = 2.381, p-value = 0.018).

Thus, miR-10b-5p may play a protective role in enhancing cell survival during stress. To model stress, we treated miRNA transfected cells with 1 uM MG 132, a potent proteasome inhibitor that increases huntingtin aggregation and cellular apoptosis in PC12 HD cell lines [27]. As expected, MG 132 treated cells had reduced cell viability as compared to untreated cells (cel-miR-67-3p, q = 6.52, adjusted p-value<0.0001; miR-10b-5p, q = 10.88, adjusted p-value<0.0001). However, MG 132 treated miR-10b-5p transfected PC12 Q73 cells exhibited improved survival over those transfected with negative control miRNA (q = 3.728, adjusted p-value = 0.045). No statistical difference was observed when comparing miR-10b-5p levels with MG 132 treatment to cel-miR-67-3p without treatment, (q = 2.95, adjusted p-value = 0.16), suggesting miR-10b-5p may enhance survival in times of cellular stress.

### miRNA expression is related to clinical variables in HD

RNA sequence count data may be non-normally distributed [28], and tests of normality for miRNA expression levels in HD found that miR-10b-5p was negatively skewed (see **Materials and Methods**). Therefore, to test the relationship of miRNA expression to clinical variables such as CAG repeat size, age at onset of motor symptoms, disease duration and age at death, as well as to the sample quality information for RIN/RQN (RNA integrity number/RNA quality number), we applied a step-wise backwards selection, negative binomial regression model.

Age at onset, duration and age at death are inter-dependent and could not be simultaneously included in the models. Furthermore, age at onset and age at death were strongly correlated with each other (Pearson r = 0.85, p-value = 5e-04) and both were correlated with CAG repeat size (r = −0.84, p-value = 6e-04, and r = −0.89, p-value = 1e-04 respectively) while duration was not correlated with age at onset, age at death or CAG repeat size in this sample. To determine which variables best modeled the relationship of the miRNAs to clinical variables, we compared the Akaike information criterion (AIC) for each variable (onset age, death age and duration) in regression analyses that adjusted for the effect of CAG repeat size. Of these three variables, duration was found to have the poorest fit with each of the five miRNAs and therefore we report analyses containing age at onset and age at death.

Among the HD brains, CAG repeat size, age at onset and age at death were all independently found to have a negative association with miR-10b-5p (CAG, β = −0.18, p-value = 2.7e-05; onset, β = −0.05, p-value = 1.9e-05; death, β = −0.07, p-value = 6.8e-07). CAG repeat size and age at onset were found to be independently, negatively related to miR-196a-5p (CAG, β = −0.15, p-value = 1.7e-02; onset, β = −0.07, p-value = 1.4e-03). Age at death was significantly related to miR-615-3p expression (β = −0.03, p-value = 0.0045) and age at onset was associated with miR-196b-5p (β = −0.04, p-value = 9e-04). No association to any clinical features was seen for miR-1247-5p. In order to fully evaluate whether there was any effect of disease duration on the observed relationships to the clinical features, duration was added back into final models. No substantial changes to the effect estimates were observed with the addition of duration to any of the models.

None of the miRNA levels was related to post-mortem interval in either control or HD case samples. The essentially null level of expression in controls prevented meaningful assessment of the relationship of miR-196a-5p, miR-196b-5p and miR-615-3p with clinical variables, in particular age at death, or sample variables, post-mortem interval (PMI), or RIN/RQN. Analysis of miR-10b-5p showed no association to age at death (β = −0.002, p-value = 0.60), or PMI (β = −0.014, p-value = 0.31), but did show association with RIN/RQN (β = 0.54, p-value = 7.2e-05) in controls. miR-1247-5p showed association with later age at death (β = −0.013, p-value = 0.024) in controls.

### Expression of miR-10b-5p, miR-196a-5p, miR-196b-5p and miR-615-3p are correlated

Among the twelve HD samples, the levels of four out of the five significantly differentially expressed miRNAs (miR-10b-5p, miR-196a-5p, miR-196b-5p, miR-615-3p) were strongly correlated with each other, (Spearman r range 0.71–0.88; p range 0.0002–0.01). miR-1247-5p was not significantly correlated with these miRNAs (Spearman r range 0.13–0.51; p range 0.09–0.70). Because the values of miR-615-3p and miR-196a-5p were essentially zero in the control samples, correlations among the miRNAs were not performed for controls.

### mRNA targets of miR-10b-5p, miR-196a-5p, miR-196b-5p and miR-615-3p may have similar functions

Watson-Crick base-pairing between nucleotide position 2 through 8 on the mature miRNA, termed the ‘seed region,’ and the 3′ untranslated region (3′ UTR) of target mRNA determine the recognition, specificity and efficiency of miRNA silencing [29]. Seed sequences differ for miR-10b-5p (ACCCUGU), miR-615-3p (CCGAGCC) and miR-1247-5p (CCCGUCC) suggesting these miRNA have different targets, while miR-196a-5p and miR-196b-5p share a seed sequence (AGGUAGU) and only differ by a single base difference in mature miRNA sequence.

Targets of the five miRNAs were obtained from miRWalk (http://www.umm.uni-heidelberg.de/apps/zmf/mirwalk/index.html), a repository of experimentally validated miRNA targets curated from literature and online resources [30]. miRWalk targets of miR-196a, miR-196b and miR-1247 were not strand specific. The miRWalk database contained 84 unique targets for miR-10b-5p, 80 for miR-196a, 40 for miR-196b, two for miR-1247 and twelve for miR-615-3p. Since miR-1247 had just two validated targets, it was removed from analysis.

Four target genes (*DICER1*, *HOXA7*, *HOXB4*, *HOXD1*) were shared across all four miRNAs. miR-10b-5p shared eleven targets with miR-196a-5p (*HOXB8*, *COX8A*, *HOXA10*, *NPC1*, *FLT3*, *AKT1*, *NPM1*, *DROSHA*, *AGO2*, *NFYC*, *PAX7*), and one with miR-615-3p (*MAPK8*). miR-196a and miR-196b shared 28 targets. In all, eleven of the 167 unique validated targets were Hox cluster genes (*HOXA1*, *HOXA7*, *HOXA9*, *HOXA10*, *HOXB4*, *HOXB7*, *HOXB8*, *HOXC8*, *HOXD1*, *HOXD4*, *HOXD10*).

To understand the influence these miRNAs may be having on shared biological processes, targets of each miRNA were analyzed using IPA Core Analysis. To find overlap in biological functions and canonical pathways of each miRNA and its targets, the IPA Core Comparison Analysis tool was used. After correcting for multiple comparisons, targets of miR-10b-5p, miR-196a, miR-196b and miR-615-3p shared significant overlap in 33 biological functions; the top three functional categories were “*Cell Death and Survival*,” (Benjamini-Hochberg adjusted p-value, range = 3.5e-07–1.5e-04), “*Nervous System Development and Function*” (range = 1.5e-07–1.5e-03) and “*Cellular Assembly and Organization*” (range = 2.5e-05–1.7e-03). Twelve pathways were shared among all four sets of miRNA targets, including “*Huntington's Disease Pathway*” (range = 7.6e-04–8.1e-03), (Gene set = *AKT1*, *BAX*, *CAPSN1*, *CLTC*, *CREB1*, *EGFR*, *HDAC9*, *JUN*, *MAPK8*).

### mRNA targets of differentially expressed miRNAs are differentially expressed

Total mRNA-sequencing was performed in the same brain samples as miRNA-sequencing to examine whether gene expression was affected by miRNA up-regulation. Of the 169 unique gene targets for the five differentially expressed miRNAs, 167 were detected using mRNA-sequencing. 22 mRNA targets were significantly differentially expressed between the HD and control prefrontal cortex samples (False Discovery Rate (FDR) adjusted q-value = 0.05 after adjusting for 167 comparisons). Only one gene (keratin 5, *KRT5*) was down-regulated in HD (**Table 4**), and four of these target genes were located in the Hox clusters (*HOXD4*, *HOXA10*, *HOXB7* and *HOXD10*).

**Table 4:** 22 differential expressed targets of miR-10b-5p, miR-196a, miR-196b, miR-1247 and miR-615-3p.

Target gene	miRNA	Location	Mean Control Expression (n = 9)	Mean HD Expression (n = 12)	Fold Change	p-value	q-value*
*SERPINE1*	miR-10b-5p	7q22.1	22.91	140.82	6.15	3.03E-11	5.06E-09
*CDKN1A*	miR-196a	6p21.2	336.73	841.75	2.5	1.58E-04	1.22E-02
*HOXD4*	miR-10b-5p	2q31.1	1.74	18.33	10.51	2.38E-04	1.22E-02
*ANXA3*	miR-10b-5p	4q21.21	259.93	553.71	2.13	2.92E-04	1.22E-02
*TWIST1*	miR-10b-5p	7p21.2	43.43	105.16	2.42	5.63E-04	1.72E-02
*CD33*	miR-196a, miR-196b	19q13.3	16.58	46.63	2.81	6.75E-04	1.72E-02
*DIO3*	miR-1247	14q32	10.89	41.93	3.85	7.29E-04	1.72E-02
*MMP2*	miR-10b-5p	16q13-q21	58.67	137.83	2.35	8.26E-04	1.72E-02
*MMP9*	miR-10b-5p	20q11.2-q13.1	5.32	17.33	3.26	9.33E-04	1.73E-02
*HOXA10*	miR-10b-5p, miR-196a, miR-196b	7p15.2	1.06	17.06	16.12	1.21E-03	1.73E-02
*RHOD*	miR-10b-5p	11q14.3	12.71	37.96	2.99	1.23E-03	1.73E-02
*COL1A1*	miR-196a	17q21.33	30.19	220.28	7.3	1.31E-03	1.73E-02
*HLA-E*	miR-10b-5p	6p21.3	3703.47	7769.76	2.1	1.34E-03	1.73E-02
*PPARA*	miR-10b-5p	22q13.31	444.7	865.02	1.95	1.53E-03	1.73E-02
*PAX6*	miR-196a	11p13	693.52	1337.23	1.93	1.62E-03	1.73E-02
*EGFR*	miR-10b-5p	7p12	784.95	1762.88	2.25	1.66E-03	1.73E-02
*HOXB7*	miR-196a	17q21.3	1.63	6.99	4.28	2.83E-03	2.78E-02
*PLAUR*	miR-10b-5p	19q13	56.15	119.67	2.13	3.65E-03	3.38E-02
*HOXD10*	miR-10b-5p	2q31.1	1.25	9.33	7.45	4.73E-03	3.96E-02
*RUNX1*	miR-10b-5p	21q22.3	87.69	224.88	2.56	4.74E-03	3.96E-02
*SOX2*	miR-10b-5p	3q26.3-q27	1963.76	3492.72	1.78	5.32E-03	4.23E-02
*KRT5*	miR-196a	12q13.13	113.74	51.99	−2.19	6.00E-03	4.55E-02

* FDR-adjusted q-value for 167 targets of the five miRNAs.

### miR-10b-5p, miR-196a-5p, miR-196b-5p and miR-615-3p expression is related to Hox cluster gene expression

Four of the five up-regulated miRNAs are located intergenic to Hox gene clusters (**Figure 3**). Because of gene duplication, miR-196a is derived from both the HOXB and HOXC clusters; miR-10b is located in the HOXD cluster and miR-615 is found in the HOXC cluster [31], [32]. A total of 55 genes (40 protein-coding genes, eleven antisense transcripts, three functional lncRNAs and one pseudogene) are located in the four Hox clusters [33], [34]. To evaluate evidence for a general regional up-regulation of Hox cluster genes, an expression analysis of the mRNA-sequence data was performed for all annotated genes within the Hox loci (**Table 5**). Fifteen out of 55 genes within the Hox loci were differentially expressed in HD. Fourteen Hox genes were significantly up-regulated (FDR-adjust q-value<0.05, mean fold-change = 6.73, range 3.02 to 16.12) and a single Hox gene was down-regulated (*HOXD1*, FDR-adjust q-value = 3.92e-02, fold change = −2.45). The majority of differentially expressed Hox genes (13 out of 15) were essentially unexpressed in controls.

**Table 5:** Differential expression of Hox cluster genes in HD.

Gene	Mean Control Expression (n = 9)	Mean HD Expression (n = 12)	Fold Change	p-value	q-value*
*HOXA11*	1.06	8.20	7.75	3.96e-05	2.07e-03
*HOXA5*	1.06	7.63	7.21	1.03e-04	2.07e-03
*HOXD8*	1.15	7.84	6.80	1.13e-04	2.07e-03
*HOXD4*	1.74	18.33	10.51	2.38e-04	3.22e-03
*HOXB9*	1.06	9.20	8.69	2.93e-04	3.22e-03
*HOXA10*	1.06	17.06	16.12	1.21e-03	1.11e-02
*HOXC6*	1.15	6.16	5.34	1.62e-03	1.27e-02
*HOXA11-AS*	1.25	7.39	5.90	2.49e-03	1.71e-02
*HOXB7*	1.63	6.99	4.28	2.83e-03	1.73e-02
*HOXA13*	1.45	8.74	6.03	4.07e-03	2.24e-02
*HOXD10*	1.25	9.33	7.45	4.73e-03	2.36e-02
*HOXD1*	55.91	22.80	−2.45	8.55e-03	3.92e-02
*HOXC10*	1.36	8.04	5.90	1.06e-02	4.14e-02
*HOXC4*	3.57	10.77	3.02	1.12e-02	4.14e-02
*HOTAIRM1*	3.52	12.52	3.56	1.13e-02	4.14e-02

* FDR-adjusted q-value for the 55 genes in the four Hox clusters.

**Figure 4:**
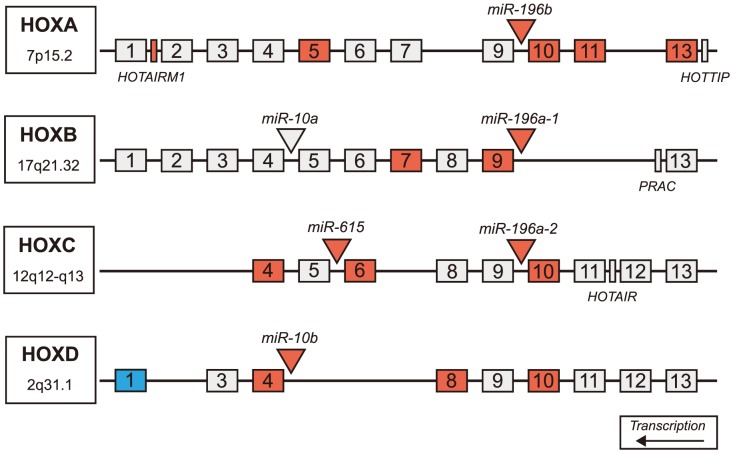
Differentially expressed miRNAs in HD are located in Hox genes clusters. Schematic representation of Hox clusters. Hox genes are represented as numbered boxes (labeled 1–13), miRNA are represented by triangles and other genes in the regions (functional lncRNA, *PRAC*) are represented by rectangles. Antisense transcripts and pseudogenes are not pictured. Nineteen genes within Hox cluster regions were found significantly differentially expressed in HD prefrontal cortex using mRNA-sequencing (FDR-adjusted p-value<0.05). Four miRNAs, one lncRNA, and fourteen Hox genes were significantly up-regulated in HD (indicated by red), many of which are adjacent to differentially expressed miRNAs. A single Hox gene (*HOXD1*) was down-regulated in HD (indicated by blue). (HD = Huntington's disease).

The genes adjacent to the four differentially expressed miRNAs were highly expressed. Two genes immediately adjacent to miR-10b-5p were significantly up-regulated in HD (*HOXD4*, FDR-adjusted q = 3.22e-03; *HOXD8*, FDR-adjusted q = 2.07e-03), (**Figure 4**). *HOXB9* (FDR-adjusted q-value = 3.22e-03) immediately downstream of miR-196a-1 and *HOXC10* (FDR-adjusted q-value = 4.14e-02) immediately upstream of miR-196a-2 were also up-regulated. Furthermore, all three Hox genes located upstream of miR-196b were significantly up-regulated in HD (*HOXA10*, FDR-adjusted q-value = 1.11e-02; *HOXA11*, FDR-adjusted q-value = 2.07e-03; *HOXA13*, FDR-adjusted q-value = 2.24e-02). *HOXC6* (FDR-adjusted q-value = 1.27e-02) immediately upstream of miR-615 was also up-regulated.

## Discussion

### Up-regulation of expression for five miRNAs in HD brain

We report a next-generation sequencing study of small RNAs, identifying 1,417 mature miRNA species in the prefrontal cortex (Brodmann Area 9) of twelve HD and nine control brains. Five of these, miR-10b-5p, miR-196a-5p, miR-196b-5p, miR-615-3p and miR-1247-5p, were up-regulated in HD at genome-wide significance (FDR q-value<0.05), and three of these five, miR-196a-5p, miR-196b-5p and miR-615-3p, were expressed at near zero levels in the control brains. Up-regulation of miR-10b-5p was validated in the miRNA-sequencing samples and confirmed in an independent replication sample set. Several studies implicating a role for miRNAs in HD have been performed, although, to our knowledge this is the first genome-wide quantification of miRNA expression comparing individual human HD and control brain samples.

Packer et al. [11], studying an array of 365 mature miRNAs, had previously reported miR-196a-5p to be significantly increased by nearly six-fold in Brodmann Area 4 of HD grade 1 brains. Recently, a study by Cheng et al. [13] found increased miR-196a expression suppressed mutant *HTT* expression in both HD neuronal cell models and HD transgenic mouse models. These findings suggest increased expression of miR-196a may be an adaptive response, promoting neuronal survival and may have therapeutic implications for HD. Miyazaki et al. [35] studied miR-196a in spinal and bulbar muscular atrophy (SBMA), a neurodegenerative disease caused by a similar polyglutamine repeat expansion in the androgen receptor (*AR*) gene. They found increased miR-196a expression via adeno-associated virus vector-mediated delivery reduced *AR* mRNA levels leading to improved neurological function in transgenic SBMA mouse models. Together, these findings suggest a neuroprotective role for miR-196a and its targets and possible therapeutic implications across multiple polyglutamine-expansion neurodegenerative diseases. miR-196a-5p and miR-10b-5p were among the 56 miRNAs found to be elevated in response to mutant *HTT* over-expression in undifferentiated NT2 cells [36]. According to the miRNA search program “PubmiR,” [37] miR-196b-5p, miR-1247-5p and miR-615-3p have not been previously reported in HD miRNA studies.

A number of past studies have examined miRNA levels in HD, HD transgenic mice or cellular models; however, we did not replicate the results obtained in these studies. Gaughwin et al. [36] reported miR-34b elevated in plasma samples in HD, but we found neither miR-34b-3p nor miR-34b-5p to be altered in HD brain at genome-wide levels. We were not able to confirm any of the miRNAs reported in past microarray studies that examined targeted subsets of miRNAs, including the nine miRNAs reported as down-regulated in two mouse models of HD (YAC128 and R6/2) by Lee et al. [14] using a 567 miRNA microarray or the 38 miRNAs with altered expression in HD transgenic mice in a 382 miRNA microarray [15]. Johnson et al. [10]–[12] reported miR-29a and miR-330 to be significantly up-regulated in HD samples, neither of which was found to be altered in this study [10]. In a RT-qPCR study comparing 90 miRNAs in mouse Hdh (Q111/Q111) striatal cells to control mice [12], [38], none of the 27 reported differentially expressed miRNAs was different at genome-wide levels in our study. The most commonly reported altered miRNA in HD studies, miR-132, has been reported as both down-regulated [10], [14], [39] and up-regulated [11], but was not differentially expressed in our study.

While some of the lack of concordance may be a consequence of the differences between human and animal models of HD, it is also likely that some of the differences are a consequence of the different technologies employed by these studies. Microarrays may have different levels of detection for some miRNAs from that seen by miRNA sequencing. Finally, nearly all of the studies employ microarray methods. Microarrays that study only 365 (e.g. Packer et al. [11],) to 567 miRNAs (e.g. Lee et al. [14]) are not performing as many contrasts and thus do not adjust for as many contrasts as our genome wide analysis (e.g. 1,417 miRNAs detected) demands.

### miR-10b-5p, miR-196a-5p, miR-196b-5p and miR-615-3p implicate Hox cluster genes

Four (miR-10b-5p, miR-196a-5p, miR-196b-5p and miR-615-3p) of the five differentially expressed miRNAs are related to Hox cluster genes as follows: (1) these four are located in intergenic regions of the Hox clusters, (2) eleven Hox genes are validated targets of these four miRNAs, (3) Hox genes adjoining differentially expressed miRNAs are differentially expressed and (4) multiple Hox cluster genes are differentially expressed in HD versus control brains (**Table 4**).

Of the eleven Hox gene targets, eight did not differ in their expression across condition. A single target, *HOXD1* was seen to be down-regulated in HD (FC = −2.45). *HOXD1* is a reported target of four of the five miRNAs [40] which may explain its repression in HD.

Three Hox gene targets were up-regulated in HD (*HOXB7*, *HOXD4 HOXD10*). It is possible these up-regulated Hox genes share similar regulatory mechanisms, as the increased miRNA expression does not produce the expected miRNA-mediated gene silencing and suppress the observed up-regulation of the miRNA target genes. Coevolution of Hox genes and Hox-related miRNAs may further suggest that they share regulatory elements or mechanisms [41]. Furthermore, Hox genes and related miRNAs have been observed to have similar patterns of transcriptional activation and both are activated by retinoic acid [42]–[46]. Although miR-10b-5p has been validated as targeting *HOXD4*, they may exhibit patterns of co-expression. Specifically, Phua et al. [45] report miR-10b and *HOXD4* are temporally co-expressed during neurodifferentiation. Here, we see a similar up-regulation and co-expression pattern in HD, where miR-10b and *HOXD4* are both highly expressed.

Hox genes are a family of transcription factors that contribute to major morphological changes during embryonic development and are required for anterior-posterior body axis in bilaterally developing species [47]. They are highly involved in most aspects of early development, and are prominently expressed in the developing brain [48]. Hox-related miRNAs may also follow similar spatio-temporal patterns of expression during embryogenesis [49].

Hox genes are regulated by retinoic acid but also other factors, including basic fibroblast growth factor [50], steroid hormones [51], [52] and polycomb repressive complex group [53]. Polycomb group (PcG) proteins assemble into large silencing complexes and control histone-modifying activity. Hox genes are repressed by PcG complexes, specifically Polycomb Repressive Complex 2 (PRC2), which trimethylates histone H3 at lysine 27 (H3K27me3) [53].

Seong et al [54] observed knockout huntingtin mouse embryos lacked repression of *HOXB1*, *HOXB2*, and *HOXB9* and showed diminished global H3K27me3, while a knock-in expanded repeat mouse exhibited increased H3K27me3 signal, suggesting mutant huntingtin may alter proper PRC2 activity. These findings raise the possibility that the increased expression of miRNAs and Hox genes reported here are related to enhanced H3K27me3 or impaired PcG repression. However, the role of Hox in the adult, HD brain is still unclear. Increased transcriptional activity of Hox may be compensatory, helping to preserve or re-establish cell polarity, or an indirect result of impaired epigenetic regulation.

### miR-10b-5p response in HD may be protective

To functionally validate our miRNA-sequencing findings, we chose to assess miR-10b-5p. We believed this miRNA to be the most biologically active of the differentially expressed miRNAs. miR-10b-5p had the highest basal expression levels and the highest fold change between conditions. Additionally, miR-10b-5p levels were not increased in PD, a comparable protein aggregate, neurodegenerative disease, nor in PD samples with pathology in the prefrontal cortex equivalent to HD.

To determine whether miR-10b-5p had a protective or deleterious effect on neuron viability, we ectopically expressed miR-10b-5p in terminally differentiated PC12 Q73 cells. Since the levels the five differentially expressed miRNA were up-regulated, we felt overexpression of miR-10b-5p best represented the phenotype observed in HD brain.

We reported increased miR-10b-5p expression enhanced the survival of PC12 Q73 cells. Furthermore, we found that increased miR-10b-5p expression enhanced survival in the presence of apoptosis-inducing compound, MG 132. In this experiment, survival in cells with increased miR-10b-5p expression was comparable to that of unchallenged cells and significantly greater than untreated cells exposed to toxin. These finding provide support for the hypothesis that increased miR-10b-5p may be a neuroprotective response to the expanded polyglutamine repeat seen in HD and speaks to the role of this microRNA in the pathology of HD.

### miR-10b-5p, miR-196a-5p, miR-196b-5p and miR-615-3p have overlapping biological functions

Using pathway analysis, we showed that miR-10b-5p, miR-196a-5p, miR-196b-5p and miR-615-3p targeted genes are predicted to be involved in apoptosis as well as nervous system development and function. In neuroblastoma SH-SY5Y cell lines, miR-10a, miR-10b and miR-615-5p expression levels significantly increased during all-trans-retinoic-acid (ATRA) treatment, indicating miR-10a/b and miR-615-5p may have a role in neurodifferentiation [44]. SH-SY5Y cells treated with antisense miR-10a or miR-10b had impaired neurite outgrowth and morphology but did not show changes in overall cell proliferation [44]. miR-10a and miR-10b were highly expressed in SK-N-BE, LAN5 and SH-SY5Y cell lines during ATRA treatment and ectopic expression of miR-10ab mirrored the phenotype of the ATRA treatment [42]. Taken together, these studies implicate these miRNAs in neuron differentiation, migration, and outgrowth.

In our past studies [16], we found increased neurite outgrowth in HD prefrontal cortex. Relative to controls, HD pyramidal neurons had a significantly increased number of primary dendritic segments, increased total dendritic length, and more dendritic branches than control neurons. Here, we report four miRNAs that have been observed in cell models to present a similar phenotype. It is possible that increased expression of these miRNAs and related targets represent an adaptive response of neurons stressed by a toxic expanded polyglutamine protein fragment.

### miR-10b-5p, miR-196a-5p, miR-196b-5p and miR-615-5p are related to HD pathogenesis

Four of the five up-regulated miRNAs showed association to clinical features of HD (CAG repeat size, age of motor onset and age at death for miR-10b-5p; CAG repeat size and age at onset for miR-196a-5p, age at onset for miR-196b-5p and age at death for miR-615-3p). Due to the near zero level of expression in controls, it was not possible to assess the relationship of miR-196a-5p, mir-196b-5p and miR-615-3p to age at death, but miR-10b-5p was not correlated with age at death in controls. Thus, the increased expression of these miRNAs did not appear to be related to normal aging, but rather a component of gene regulation and transcription in the context of neurodegeneration. A growing body of literature points to the presence of toxic effects of the HD gene substantially before the onset of symptoms, perhaps from the time of conception [55]–[57].

Because age at death represents the lifetime exposure of the individual to the effects of the HD gene, we hypothesize that the association of miR-10b-5p and miR-615-3p with age at death may represent the lifetime exposure to the effects of the HD mutation. If the relationship of altered miRNA expression to age at death supports the view that the HD gene may have a life-long effect among expanded CAG-repeat carriers, this raises the possibility that the HD mutation may influence neuronal development in the developing brain through the action of one or more of these miRNAs and Hox cluster genes.

### Target genes of over-expressed miRNAs show increased expression in HD

We report five miRNAs as being highly up-regulated in HD and though our expectation was to see the mRNA targets of these miRNAs as decreased, we observe increased expression of many of their shared mRNA targets. We believe these effects are not attributable to differences in cell populations studied, since flow cytometric analysis measuring neuron abundance found no significant difference across condition. Rather, we hypothesize positive miRNA-mRNA target relationships are a result of HD-specific alterations in mRNA processing.

Translation is a highly dynamic process. Cytoplasmic mRNA actively engaged in translation can cycle to a non-translated state and accumulate in stress granules or processing bodies (P-bodies). During cellular stress, mRNA can be sequestered to P-bodies or stress granules, to stall translation through translational repression machinery or miRNA silencing, until stress conditions have been resolved [7], [58]–[60]. P-bodies may also serve an important role in RNA transport. Because neurons are highly polarized, cytoplasmic transport of mRNA is essential for localized translation to discrete regions of the cell. During transport, it is believed that mRNAs are silenced by miRNA, upon rapid exchange at the synapse [60]–[62].

In HD cortical neurons, excitotoxicity, oxidative damage, aberrant gene expression and energetic defects lead to stress conditions and in response, cells may sequester mRNA to P-bodies and stress granules. Among the 55 Hox locus genes studied, only one of the fifteen significantly differentially expressed genes is down regulated (**Table 4**). Thus, the increased levels of most of the validated gene targets of these four miRNAs may be reactionary, as they are sequestered to P-bodies for storage as part of a protective process to enhance cell viability [7].

To the best of our knowledge, no study has addressed the role of P-bodies or stress granules in HD. However, it was observed in live cortical neurons that wildtype huntingtin co-localized in P-bodies, specifically in neuronal RNA granules, along with Argonaute 2, the endonuclease required for RNA-mediated gene silencing by the RNA-induced silencing complex (RISC) [63], [64]. Therefore, it is reasonable to suggest mutant huntingtin may impair miRNA-mediated mRNA degradation and/or localized translation of specific mRNAs.

There is evidence that miRNA-mRNA regulatory mechanisms may be altered in other neurodegenerative diseases as well. In a joint examination of miRNA-mRNA expression in Alzheimer's disease (AD) and control prefrontal cortex, an overwhelming number of miRNA to mRNA targets were found to be positive correlated. Genomic variants in TDP-43 and FUS, genes that encode stress granule proteins, were found to cause familial Amyotrophic lateral sclerosis [65], [66] and several other stress granule proteins (TIA-1, G3BP) may also be pathogenic [67].

### miRNAs as potential biomarkers in HD

These studies suggest potential relationships of these miRNAs to CAG repeat expansion, age at onset or age at death. If these findings hold up on further examination, these miRNAs may hold potential to provide insight into important biological and disease expression for HD. miRNA are extremely stable. The half-life of the majority of miRNAs has been predicted to be on average five days and plasma miRNAs have been found to be stable after being subjected to high heat, extreme pH, long-time storage at room temperature, or multiple freeze-thaw cycles [68]–[70]. If these miRNAs cross the blood-brain barrier and can be detected at reasonable levels in serum/plasma from mutant HD gene carriers, they may serve as biomarkers of disease expression.

## Materials and Methods

### Sample information

Frozen brain tissue from prefrontal cortex Brodmann Area 9 (BA9) was obtained from the Harvard Brain and Tissue Resource Center (HBTRC) McLean Hospital, Belmont MA. Twelve Huntington's disease (HD) samples and eleven neurologically-normal control samples were selected for the study (**Table 1**). The HD subjects had no evidence of Alzheimer or Parkinson disease (PD) comorbidity based on neuropathology reports. For microscopic examination, 16 tissue blocks were systematically taken and histologically assessed as previously described [3]. All samples were male. HD samples and controls were not different for postmortem interval (PMI) (t = 1.07, p = 0.30), RNA integrity number (RIN) (t = 0.83, p = 0.41) or death age (t = 0.40, p = 0.69). CAG repeat size was known for all HD samples and onset age and disease duration was unknown for a single sample (**Table 1**). Eight additional HD, nine control and fourteen PD cases were studied as part of validation and replication studies, and were obtained from the HBTRC and the Sun Health Research Institute Sun City, Arizona (**Tables S2, S3 and S5**).

### RNA extraction

Total RNA, for all samples studied, was isolated using QIAzol Lysis Reagent and purified using miRNeasy MinElute Cleanup columns (Qiagen Sciences Inc, Germantown, MD). RNA quality for sequencing was assessed using either Agilent's BioAnalyzer 2100 system and RNA 6000 Nano Kits to find RNA Integrity Number (RIN) or Agilent 2200 TapeStation and DNA ScreenTape assay RNA Quality Number (RQN; Agilent, Foster City, CA). Both methods calculate the area under the peak for 18S and 28S RNA as a ratio of total RNA as well as the relative height of the 18S and 28S peaks to determine RNA quality [71]. The RIN/RQN values were similar for the twelve HD and eleven control specimens studied for miRNA and mRNA (t = 0.95, p = 0.36).

### Illumina miRNA sequencing (miRNA-seq)

For each brain sample, 1 ug of RNA was used to construct sequencing libraries using Illumina's TruSeq Small RNA Sample Prep Kit, according to the manufacturer's protocol (Illumina, San Diego, CA). In brief, small RNA molecules were adapter-ligated, reverse transcribed, PCR amplified and gel purified to generate the library. Multiplexed samples were equimolarly pooled into sets of eight samples per flowcell lane and sequenced using 1×50 bp single-end reads on Illumina's HiSeq 2000 system at Tufts University sequencing core facility (http://tucf-genomics.tufts.edu/). Demultiplexing and FASTQ file generation (raw sequence read plus quality information in Phred format) were done using Illumina's Consensus Assessment of Sequence and Variation (CASAVA) pipeline.

### Primary processing of Illumina miRNA-seq reads

Sequence read quality was evaluated using the FASTQ quality filter module from the FASTX-toolkit version 0.0.13 (http://hannonlab.cshl.edu/fastx_toolkit/), and only those reads with at least 80% of the base calls above Q20 (Phred score) were retained. The 3′ adapter sequence (5′-TGGAATTCTCGGGTGCCAAGG-3′) was removed from all reads using the FASTA/Q clipper module from the FASTX-toolkit. A minimum length threshold of 15 nucleotides was set for clipped reads because miRNAs of this length will contain the seed sequence. To avoid redundancy amongst identical read species, the reads were collapsed using the FASTA/Q collapser module from FASTX-toolkit to generate a FASTA file of only the unique read species.

### Alignment and mapping of miRNA-seq reads

Quality-filtered, 3′ adapter-clipped reads were aligned to the UCSC human reference genome (build hg19) using Bowtie version 0.12.3 [72]. Alignment parameters were set to allow for no mismatch alignments and no limits on multiple mapping instances. Multiple-mapped identical sequences were summed for a single count for that annotated mature miRNA. The default settings were used for all other alignment options.

The miRNA aligned data are available on ArrayExpress (http://www.ebi.ac.uk/arrayexpress/), accession number E-MTAB-2206.

### miRNA abundance estimation

Aligned reads that overlapped with the human miRNA annotation version 19 from miRBase (http://www.mirbase.org/ftp.shtml) were identified using default BEDTools' *IntersectBed* functionality [73]. To select for mature miRNA reads, sequences more than 27 bases in length were removed. Only those reads for which the aligned 5′ start-nucleotide matched exactly to the 5′ start-nucleotide of the annotated miRNA were retained for the analysis. After filtering, collapsed read counts were summed per annotated mature miRNA (**Table S6**).

### miRNA differential expression

The R (http://www.R-project.org) package DESeq version 1.10.1 [28] was used to perform the differential expression analysis between HD and control samples using the read counts generated for each sample as described above. miRNAs with zero read counts across all case and control samples were removed from analysis. To accommodate the analysis of miRNAs with read counts of zero for some samples, a pseudo-count of one was added to all raw counts for every miRNA across all the samples, prior to performing DESeq's estimateSizeFactors and estimateDispersions functions with default options. DESeq assumes that count data follow a negative binominal distribution and factors in technical and biological variance when testing for differential gene expression between groups. DESeq's function, estimateSizeFactors, was used to obtain normalization factors for each sample and to normalize miRNA read counts.

The normalized counts were evaluated by principal component analysis (PCA) with the FactoMineR R package for all HD and control samples. The samples identified to be three or more standard deviations away from the mean on the first or second principal component were considered outliers and were removed from analysis. The first two principal components were used because they each explained more than 10% of the variance, while the remaining principal components explained less than 10% of the variance. Two control samples (C-35 and C-37) were identified as outliers based on PCA analysis.

miRNA differential expression analysis was performed with DESeq's nbinomTest function for the remaining nine control and twelve HD samples. All analyses were performed on DESeq normalized counts.

### miRNA quantitative PCR

miRNA were assayed using Exiqon's miRCURY LNA Universal RT miRNA PCR following the manufacturer's protocol (Exiqon Inc, Denmark). In brief, reactions were incubated for 60 min at 42°C followed by heat-inactivation of reverse transcription for 5 min at 95°C and stored at 4°C. After cDNA synthesis, samples were diluted to 0.2 ng/ul in water. Brain samples were assayed using Exiqon ExiLENT SYBR Green master mix and LNA primer sets containing UniRT and miR-10b-5p, miR-196a-5p, miR-196b-5p, miR-615-3p or miR-1247. Reference primer hsa-SNORD48 PCR/UniRT was used for brain samples; U6 snRNA for cell lines. Samples were run in triplicate for each primer set in 384-well format (5 ul PCR Master mix, 1 ul PCR primer mix, 4 ul 0.2 ng cDNA). Reactions were cycled using Applied Biosystems 7900HT Fast Real-Time PCR System using manufacturer's instructions (Life Technologies, Carlsbad, CA). For analysis, threshold cycle (C_T_) was generated by ABi SDS v2.4 software. C_T_ values for triplicate wells were normalized by average RNU48 value for brain or U6 for cells. miRNA fold change was calculated using the 2-ΔΔCT method [74].

### Neuron abundance quantification

0.5–1.0 g of tissue in 5 ml of lysis buffer was homogenized using a dounce tissue grinder. Lysates were transferred to ultracentrifugation tubes, loaded on top of sucrose solution and centrifuged at 24,400 RPM for 2.5 hr at 4°C (Beckman Coulter, Pasadena, CA; L8-70 M with SW80 rotor). Nuclei pellets were resuspended in 500 ul PBS and incubated at 4°C in a staining solution containing 0.72% normal goat serum, 0.036% BSA, 1∶1200 anti-NeuN (Millipore, Germany), 1∶1400 Alexa488 goat anti-mouse secondary antibody (Life Technologies, Carlsbad, CA), for 45 min. Flow cytometry was performed at the Boston University Medical School Flow Cytometry Core Lab on a FACSVantage SE flow cytometer.

### Illumina messenger RNA sequencing (mRNA-seq)

For each brain sample, 1 ug of RNA was used to construct sequencing libraries using Illumina's TruSeq RNA Sample Prep Kit according to the manufacturer's protocol. In brief, mRNA molecules were polyA selected, chemically fragmented, randomly primed with hexamers, synthesized into cDNA, 3′ end-repaired and adenylated, sequencing adapter ligated and PCR amplified. Each adapter-ligated library contained one of twelve TruSeq molecular barcodes. Multiplexed samples were equimolarly pooled into sets of three samples per flowcell lane and sequenced using 2×100 bp paired-end reads on Illumina's HiSeq 2000 system at Tufts University sequencing core facility (http://tucf-genomics.tufts.edu/). Demultiplexing and FASTQ file generation were accomplished using Illumina's CASAVA pipeline.

### Primary processing of Illumina mRNA-seq reads

Forward and reverse sequencing reads were independently quality-filtered using the FASTQ quality filter module from the FASTX-toolkit version 0.0.13 (http://hannonlab.cshl.edu/fastx_toolkit/) with the same criteria as that applied for the processing of the miRNA-seq reads. Reads failing the quality threshold, as well as their corresponding mate reads, were removed.

### Alignment and mapping of mRNA-seq reads

Quality-filtered paired-end reads were aligned to the UCSC human reference genome (build hg19) using TopHat version 2.0.4 [75], [76]. This version of TopHat incorporates the Bowtie version 2.0.0.7 algorithm to perform the alignment [72] as well as SAMtools version 0.1.18.0 for alignment file formatting [77]. For efficient read mapping, TopHat requires the designation of the mean and standard deviation of the distance between paired-end reads, the read inner-distance. To estimate the appropriate read inner-distance, we aligned a subset of 5 million reads from four HD and four control samples to the Ensembl human reference transcriptome (release 66) using Bowtie version 2.0.0.7. Using the CollectInsertSizeMetrics function from picardTools version 1.76 (http://sourceforge.net/projects/picard/files/picard-tools/), we estimated the average mean inner-distance per condition and subsequently applied these values for the TopHat alignment; 22 for HD samples 25 for controls respectively, (the current TopHat default setting is 20), (**Table S7**). To account for read variability, the standard deviation for inner-distance was set to 100. The number of allowed splice mismatches was set to 1. Default settings were used for all other alignment options.

### mRNA gene abundance estimation

Gene expression quantification was performed using htseq-count version 0.5.3p9 (http://www-huber.embl.de/users/anders/HTSeq) and the GENCODE version 14 annotation gtf file as reference (http://www.gencodegenes.org/releases). Intersection non-empty mode and unstranded library type were specified as parameters for htseq-count. Default settings were used for all other options (**Table S8**).

### mRNA differential expression analysis

The mRNA differential expression analysis between HD and control samples was performed using DESeq version 1.10.1 [28]; the workflow was the same as described for the miRNA differential expression analysis. No outliers were found based on the PCA of the DESeq-normalized count data. The nbinomTest function was run for eleven control samples and twelve HD samples to assess differentially expressed genes. Multiple comparison adjustment for multiple testing with the Benjamini-Hochberg correction was used to control for false discovery rate. For Hox gene differential expression analysis, 55 comparisons were used. Genes located within HOX-gene containing regions were queried through the Ensembl database (release 72), interfacing through the R package BiomaRt [78], [79]. Genes that were between *HOXA1-HOXA13*, *HOXB1-HOXB13*, *HOXC4-HOXC13* and *HOXD1-HOXD13* start sites were regarded as “Hox genes.” For miRNA target differential expression, 154 comparisons were used for Benjamini-Hochberg correction.

### miRNA-mRNA target analysis

Information on experimentally validated miRNA targets of miR-10b-5p, miR-196a-5p and miR-615-3p were extracted from the miRWalk “Validated Targets” module [30]. Strand specificity was preserved. Targets for miR-196a-1 and miR-196a-2 were merged for analysis. IPA Core Analysis (analysis.ingenuity.com) was run as nervous system and CNS cell line specific across all species, using target gene lists imported from miRWalk output. “Bio Functions” and “Canonical Pathway” analyses were used. Right-tailed Fisher's Exact Tests were run through IPA software and p-values with FDR-adjusted q-values (p<0.05) were considered significant. Biological functions across the 3 significant miRNA were compared using the IPA Core Comparison Analysis tool. Benjamini-Hochberg Multiple Testing Correction p-values (p<0.05) were considered significant.

### Linear modeling of miRNA relationship to clinical covariates

To account for the non-normality in the miRNA data, negative binominal general linear regressions were performed using Proc genmod in SAS. DESeq normalized counts were rounded to the nearest integer before running the model. To test the normality of gene expression data, Shapiro-Wilk tests were performed. Differentially expressed miRNA data trended as non-normally distributed in HD (miR-10b-5p, p = 0.04; miR-196a-5p, p = 0.05; miR-615-3p, p = 0.06), but not in controls (miR-10b-5p, p = 0.71; miR-196a-5p and miR-615-3p were essential zero).

### Generation of transgenic cell lines

PC12 (rat adrenal gland phaeochromocytoma) cells were grown at 37°C and 5% CO_2_ in Dulbecco's modified Eagle's medium (DMEM; Life Technologies, Carlsbad, CA) with 20% fetal bovine serum (FBS; Atlanta Biologicals, Flowery Branch, GA), 100 units/ml penicillin and 100 units/ml streptomycin (Life Technologies, Carlsbad, CA). pcDNA3.1mycC expressing human huntingtin fragment (1–90) containing 73 polyglutamine repeats (Coriell Institute; CHDI-90000034) was used for stable transfection. Cells were seeded to 70% confluency and grown overnight. 15 µl of Attractene Transfection Reagent (Qiagen, Gaithersburg, MD) was added to 4 µg plasmid DNA diluted in 300 µl Opti-MEM (Life Technologies, Carlsbad, CA). Cells were grown in complete media and selected for four weeks using 500 mg/ml G418 (Life Technologies, Carlsbad, CA). To create monoclonal cultures, single colonies were isolated using dilution cloning, picked with filter paper, grown in a 6-well plate and screened for transgenic expression by Western blot analysis using mouse Anti- c-Myc (Novex, R950-25, Life Technologies, Carlsbad, CA).

### Cell differentiation and miRNA overexpression

96-well culture plates were seeded with 10,000 cells per well. For differentiation, culture medium was replaced with medium composed of DMEM with 0.5% FBS, 100 mg/ml G418, 100 units/ml penicillin and 100 units/ml streptomycin and 100 ng/ml nerve growth factor (R&D Systems, Minneapolis, MN). After 48 hr, miRNA was transfected into HD cells using 0.25 ul Lipofectamine 2000 (Life Technologies, Carlsbad, CA) and 6.25 pmol miR-10b-5p or miRIDIAN microRNA Mimic Negative Control #1 (cel-miR-67-3p, Thermo Scientific, Waltham, MA) per well, following manufacturer's protocol. miR-10b-5p overexpression was verified using qPCR.

### Cell viability assays

For MTT assays, 1 uM MG 132 (Tocris Bioscience, United Kingdom) was added to select wells containing 10,000 cells per well at 72 hr post-differentiation. Cell viability was assessed at 96 hr post-differentiation. Following manufacturer's protocol, CellTiter 96 Non-Radioactive Cell Proliferation Assay kit (Promega; Madison, WI) was used to determine cell number. Cells were incubated for 1.5 hr at 37°C and 5% CO_2_ with MTT dye solution. Undifferentiated HD cells were serially diluted across a 96-well plate to create a standard curve for cell number calculation. Absorbance was measured using Bio-Tek Synergy H1 spectrophotometer at 540 nm for miR-10b-5p transfected wells, with MG 132 (n = 44) and without MG 132 (n = 35) and cel-miR-67-3p transfected wells with MG 132 (n = 40) and without MG 132 (n = 40). One-way ANOVA way used for statistical analysis.

For cell viability staining, miR-10b-5p and negative control mimic were transfected after 48 hours of differentiation in 12-well culture plate with 4 replicates each, 250,000 cells per well. Molecular Probes Neurite Outgrowth Staining Kit (Life Technologies, Carlsbad, CA) was used according to manufacturer's protocol. Using Bio-Tek Synergy H1 microplate reader, fluorescent area scans were taken at 530 nm excitation/590 nm emission with a 5×5 matrix per well.

## Supporting Information

Figure S1 Neuron counts from prefrontal cortical tissue homogenate. No significant difference is observed when comparing ratios of NeuN+ counts to total events quantified by flow cytometry.
(EPS)

Table S1 miRNA RT-qPCR validation study results. RT-qPCR was used to validate the five differentially expressed miRNA in the same set of sample used for miRNA-sequence analysis. The table lists the difference and standard error of fold change between condition (2-ΔΔCt), as well as p-values from two-tailed Welch's t-tests, for ten control and eleven Huntington's disease (HD) samples.
(DOCX)

Table S2 Sample information for eight Huntington's disease brains used for RT-qPCR replication study. Post-mortem intervals (PMI), RNA integrity numbers (RIN) and ages at death for the eight Huntington's disease (HD) brains used for RT-qPCR verification of the five differentially expressed miRNA.
(DOCX)

Table S3 Sample information for eight control brains used for RT-qPCR replication study. Post-mortem intervals (PMI), RNA integrity numbers (RIN) and ages at death for the eight control brains used for RT-qPCR verification of the five differentially expressed miRNA.
(DOCX)

Table S4 miRNA RT-qPCR replication study results. RT-qPCR was used to replicate the five differentially expressed miRNA in an independent sample set of Huntington's disease (HD) brains. The table lists the difference and standard error of fold change between condition (2-ΔΔCt), as well as p-values from one-tailed Welch's t-tests, for eight control and eight Huntington's disease samples.
(DOCX)

Table S5 Sample information for fourteen Parkinson's disease brains used for RT-qPCR replication study. Post-mortem intervals (PMI), RNA integrity numbers (RIN) and ages at death for the fourteen Parkinson's disease (PD) brains used for RT-qPCR verification of the five differentially expressed miRNA.
(DOCX)

Table S6 Read statistics for miRNA-sequence analysis. Summary of Illumina miRNA-sequence read and quality control statistics generated from the FASTX-toolkit.
(DOCX)

Table S7 Mean and standard deviation inner-distance estimates for TopHat2 alignment. Statistics used to estimate the distance between paired-end reads, generated from picardTools.
(DOCX)

Table S8 Read statistics for mRNA-sequence analysis. Summary of Illumina mRNA-sequence read and quality control statistics generated from the FASTX-toolkit.
(DOCX)
