# Supplementary material for: MicroRNAs Located in the Hox Gene Clusters Are Implicated in Huntington's Disease Pathogenesis
Source: PLoS Genet. 2014 Feb 27;10(2):e1004188. doi: 10.1371/journal.pgen.1004188 (PMC3937267; doi:10.1371/journal.pgen.1004188)
Supplement: Table S1 — miRNA RT-qPCR validation study results. RT-qPCR was used to validate the five differentially expressed miRNA in the same set of sample used for miRNA-sequence analysis. The table lists the difference and standard error of fold change between condition (2-ΔΔCt), as well as p-values from two-tailed Welch's t-tests, for ten control and eleven Huntington's disease (HD) samples. (DOCX) [file pgen.1004188.s002.docx]

Table S1: miRNA RT-qPCR validation study results

| miRNA | Difference between mean fold change | p-value |
| --- | --- | --- |
| miR-10b-5p | 59.20 ± 12.52 | <0.0001 |
| miR-196a-5p | 23.41 ± 6.960 | 0.0008 |
| miR-196b-5p | 6.138 ± 1.464 | 0.0072 |
| miR-615-3p | 6.808 ± 1.615 | 0.0016 |
| miR-1247 | 1.337 ± 0.3868 | 0.004 |
