# Supplementary material for: MicroRNAs Located in the Hox Gene Clusters Are Implicated in Huntington's Disease Pathogenesis
Source: PLoS Genet. 2014 Feb 27;10(2):e1004188. doi: 10.1371/journal.pgen.1004188 (PMC3937267; doi:10.1371/journal.pgen.1004188)
Supplement: Table S2 — Sample information for eight Huntington's disease brains used for RT-qPCR replication study. Post-mortem intervals (PMI), RNA integrity numbers (RIN) and ages at death for the eight Huntington's disease (HD) brains used for RT-qPCR verification of the five differentially expressed miRNA. (DOCX) [file pgen.1004188.s003.docx]

Table S2: Sample information for eight Huntington’s disease brains used for hsa-miR-10b-5p RT-qPCR replication study

| ID | PMI | RIN | Death age | Onset age | CAG Repeat Size | Duration | Neuron Loss in Neocortical Gray Matter |
| --- | --- | --- | --- | --- | --- | --- | --- |
| HD-539 | 14.5 | 6.5 | 54 | 42 | 45 | 12 | 0 |
| HD-657 | 24.3 | 8.1 | 61 | 36 | 45 | 25 | 2 |
| HD-658 | 11 | 7.8 | 48 | 42 | 44 | 6 | 1 |
| HD-681 | 19.06 | 7 | 69 | 50 | 42 | 19 | 1 |
| HD-695 | 16.15 | 7.9 | 55 | NA | 45 | NA | 1 |
| HD-700 | 15.66 | 8 | 50 | 33 | 47 | 17 | 1 |
| HD-709 | 7.1 | 8.1 | 51 | 45 | 45 | 6 | NA |
| HD-726 | 14.75 | 9.2 | 50 | 27 | 48 | 23 | 1 |
| Mean | 15.32 | 7.83 | 54.75 | 39.29 | 45.13 | 15.43 | 1 |

Scale of neuron loss: 0=absent, 1=mild, 2=moderate
