# Supplementary material for: MicroRNAs Located in the Hox Gene Clusters Are Implicated in Huntington's Disease Pathogenesis
Source: PLoS Genet. 2014 Feb 27;10(2):e1004188. doi: 10.1371/journal.pgen.1004188 (PMC3937267; doi:10.1371/journal.pgen.1004188)
Supplement: Table S3 — Sample information for eight control brains used for RT-qPCR replication study. Post-mortem intervals (PMI), RNA integrity numbers (RIN) and ages at death for the eight control brains used for RT-qPCR verification of the five differentially expressed miRNA. (DOCX) [file pgen.1004188.s004.docx]

Table S3: Sample information for eight control brains used for hsa-miR-10b-5p RT-qPCR replication study

| ID | PMI | RIN | Death |  |  |  |
| --- | --- | --- | --- | --- | --- | --- |
| C-12 | 19 | 7.1 | 66 |  |  |  |
| C-13 | 15 | 7.8 | 69 |  |  |  |
| C-15 | 10 | 8.2 | 61 |  |  |  |
| C-16 | 20 | 8.4 | 58 |  |  |  |
| C-17 | 21 | 8.2 | 70 |  |  |  |
| C-18 | 17 | 8.5 | 66 |  |  |  |
| C-20 | 20 | 7.9 | 60 |  |  |  |
| C-70 | 19 | 6.3 | 68 |  |  |  |
| Mean | 17.6 | 7.8 | 65.8 |  |  |  |
