# Supplementary material for: MicroRNAs Located in the Hox Gene Clusters Are Implicated in Huntington's Disease Pathogenesis
Source: PLoS Genet. 2014 Feb 27;10(2):e1004188. doi: 10.1371/journal.pgen.1004188 (PMC3937267; doi:10.1371/journal.pgen.1004188)
Supplement: Table S4 — miRNA RT-qPCR replication study results. RT-qPCR was used to replicate the five differentially expressed miRNA in an independent sample set of Huntington's disease (HD) brains. The table lists the difference and standard error of fold change between condition (2-ΔΔCt), as well as p-values from one-tailed Welch's t-tests, for eight control and eight Huntington's disease samples. (DOCX) [file pgen.1004188.s005.docx]

Table S4: miRNA RT-qPCR replication study results

| miRNA | Difference between mean fold change | p-value |
| --- | --- | --- |
| miR-10b-5p | 18.96 ± 3.888 | 0.0009 |
| mir-196a-5p | 8.390 ± 1.985 | 0.0029 |
| mir-196b-5p | 3.710 ± 1.788 | 0.0382 |
| miR-615-3p | 2.153 ± 1.147 | 0.0453 |
| miR-1247 | 0.8107 ± 0.5077 | 0.0684 |
