# Supplementary material for: MicroRNAs Located in the Hox Gene Clusters Are Implicated in Huntington's Disease Pathogenesis
Source: PLoS Genet. 2014 Feb 27;10(2):e1004188. doi: 10.1371/journal.pgen.1004188 (PMC3937267; doi:10.1371/journal.pgen.1004188)
Supplement: Table S5 — Sample information for fourteen Parkinson's disease brains used for RT-qPCR replication study. Post-mortem intervals (PMI), RNA integrity numbers (RIN) and ages at death for the fourteen Parkinson's disease (PD) brains used for RT-qPCR verification of the five differentially expressed miRNA. (DOCX) [file pgen.1004188.s006.docx]

Table S5: Sample information for fourteen Parkinson’s disease (PD) brains used for hsa-miR-10b-5p RT-qPCR replication study

| ID | PMI | Death | Cortical Gliosis in Neocortical Gray Matter | Neuron Loss in Neocortical Gray Matter |
| --- | --- | --- | --- | --- |
| PD-012 | 2 | 69 | NA | NA |
| PD-023 | 27 | 80 | 2 | 2 |
| PD-034 | 4 | 64 | NA | NA |
| PD-062 | 3 | 88 | NA | NA |
| PD-092 | 14 | 67 | 0 | 2 |
| PD-101 | 26 | 77 | 1 | 1 |
| PD-102 | 21 | 83 | 1 | 1 |
| PD-111 | 18 | 73 | 0 | 0 |
| PD-126 | 30 | 75 | 0 | 0 |
| PD-130 | 18 | 68 | 0 | 0 |
| PD-132 | 16 | 95 | 0 | 0 |
| PD-133 | 23 | 74 | 0 | 0 |
| PD-138 | 19 | 75 | 1 | 1 |
| PD-139 | 11 | 77 | 1 | 2 |
| Mean | 16.57 | 76.07 | 0.55 | 0.82 |
