# Supplementary material for: MicroRNAs Located in the Hox Gene Clusters Are Implicated in Huntington's Disease Pathogenesis
Source: PLoS Genet. 2014 Feb 27;10(2):e1004188. doi: 10.1371/journal.pgen.1004188 (PMC3937267; doi:10.1371/journal.pgen.1004188)
Supplement: Table S6 — Read statistics for miRNA-sequence analysis. Summary of Illumina miRNA-sequence read and quality control statistics generated from the FASTX-toolkit. (DOCX) [file pgen.1004188.s007.docx]

Table S6: Read statistics for miRNA-sequence analysis

| Sample name | Raw reads | Reads passing quality filtering | Reads after Illumina small RNA 3’ adapter clipping (L >= 15) | No. unique sequences | Reads aligned to 5' start-base of mature miRNAs |
| --- | --- | --- | --- | --- | --- |
| C-14 | 17,874,597 | 17,702,304 | 17,490,548 | 1,228,711 | 9,963,825 |
| C-21 | 17,001,863 | 16,884,322 | 15,697,016 | 1,986,629 | 5,617,765 |
| C-29 | 16,888,582 | 16,791,603 | 15,467,977 | 999,540 | 5,875,221 |
| C-31 | 20,121,708 | 19,921,480 | 18,922,152 | 1,849,706 | 12,278,125 |
| C-32 | 17,980,268 | 17,800,894 | 17,274,998 | 832,059 | 10,903,048 |
| C-33 | 23,457,233 | 23,220,267 | 21,747,546 | 1,579,663 | 15,253,737 |
| C-35 | 85,321,866 | 82,781,727 | 58,259,320 | 5,506,158 | 23,941,470 |
| C-36 | 21,339,492 | 21,162,821 | 20,488,794 | 1,100,254 | 11,844,883 |
| C-37 | 119,039,780 | 117,415,277 | 116,127,648 | 8,212,448 | 72,666,381 |
| C-38 | 35,988,842 | 35,717,601 | 35,449,135 | 994,045 | 10,253,581 |
| C-39 | 17,706,807 | 17,573,521 | 16,812,045 | 597,200 | 9,872,223 |
| HD-01 | 17,436,483 | 17,264,732 | 16,622,023 | 1,939,784 | 10,017,785 |
| HD-02 | 21,069,321 | 20,853,553 | 20,076,336 | 558,786 | 15,188,076 |
| HD-03 | 17,482,548 | 17,306,462 | 16,608,972 | 421,421 | 16,281,799 |
| HD-05 | 20,276,057 | 20,100,932 | 19,799,908 | 387,984 | 6,919,180 |
| HD-06 | 14,545,643 | 14,461,655 | 13,962,076 | 528,006 | 8,070,046 |
| HD-07 | 13,606,125 | 13,534,228 | 12,671,886 | 550,458 | 8,586,199 |
| HD-08 | 19,266,661 | 19,161,989 | 18,087,895 | 521,311 | 10,402,583 |
| HD-09 | 21,426,693 | 21,304,171 | 20,125,320 | 554,417 | 14,029,994 |
| HD-10 | 20,683,130 | 20,538,776 | 20,231,810 | 714,336 | 18,482,773 |
| HD-12 | 20,906,712 | 20,748,485 | 20,457,694 | 296,719 | 6,938,691 |
| HD-13 | 23,610,018 | 23,408,099 | 23,172,857 | 1,691,514 | 8,843,106 |
| HD-14 | 14,285,476 | 14,164,185 | 13,805,549 | 461,081 | 10,743,948 |
| Mean | 26,839,822 | 26,513,873 | 24,754,761 | 1,457,053 | 14,042,367 |
