# Supplementary material for: MicroRNAs Located in the Hox Gene Clusters Are Implicated in Huntington's Disease Pathogenesis
Source: PLoS Genet. 2014 Feb 27;10(2):e1004188. doi: 10.1371/journal.pgen.1004188 (PMC3937267; doi:10.1371/journal.pgen.1004188)
Supplement: Table S7 — Mean and standard deviation inner-distance estimates for TopHat2 alignment. Statistics used to estimate the distance between paired-end reads, generated from picardTools. (DOCX) [file pgen.1004188.s008.docx]

Table S7: Mean and std. deviation inner-distance estimates for TopHat2 alignment

| Sample | Mean inner-distance | Std* inner-distance | Mean inner-distance |
| --- | --- | --- | --- |
| C-14 | 29 | 46.57 | **25** |
| C-29 | 22 | 44.88 |  |
| C-33 | 23 | 45.44 |  |
| C-35 | 25 | 46.16 |  |
| HD-03 | 27 | 45.04 | **22** |
| HD-05 | 25 | 44.14 |  |
| HD-07 | 20 | 47.59 |  |
| HD-14 | 17 | 46.06 |  |
