# Supplementary material for: MicroRNAs Located in the Hox Gene Clusters Are Implicated in Huntington's Disease Pathogenesis
Source: PLoS Genet. 2014 Feb 27;10(2):e1004188. doi: 10.1371/journal.pgen.1004188 (PMC3937267; doi:10.1371/journal.pgen.1004188)
Supplement: Table S8 — Read statistics for mRNA-sequence analysis. Summary of Illumina mRNA-sequence read and quality control statistics generated from the FASTX-toolkit. (DOCX) [file pgen.1004188.s009.docx]

Table S8: Read statistics for mRNA-sequence analysis

| Sample ID | Reads passing quality filtering (R1) | Reads passing quality filtering (R2) | Paired reads passing quality filtering (R1 \| R2) | Alignment rate (R1) | Alignment rate (R2) |
| --- | --- | --- | --- | --- | --- |
| C-14 | 30,967,563 (95%) | 30,723,358 (94%) | 29,938,083 (92%) | 90.62% | 90.50% |
| C-21 | 38,132,322 (96%) | 37,619,888 (95%) | 36,881,921 (93%) | 90.32% | 90.23% |
| C-29 | 28,174,695 (95%) | 27,802,226 (94%) | 27,237,010 (92%) | 92.13% | 92.04% |
| C-31 | 34,276,212 (94%) | 33,994,980 (93%) | 33,080,970 (90%) | 91.31% | 91.18% |
| C-32 | 33,604,808 (95%) | 33,077,499 (94%) | 32,372,470 (91%) | 89.02% | 88.93% |
| C-33 | 33,185,676 (96%) | 32,699,140 (95%) | 31,988,954 (92%) | 91.10% | 91.03% |
| C-35 | 29,925,583 (97%) | 29,563,266 (95%) | 28,998,262 (93%) | 88.52% | 88.44% |
| C-36 | 30,216,201 (95%) | 29,781,333 (94%) | 29,169,093 (91%) | 90.46% | 90.36% |
| C-37 | 29,781,333 (94%) | 28,907,690 (96%) | 27,877,703 (87%) | 90.32% | 90.21% |
| C-38 | 28,368,133 (93%) | 27,900,649 (92%) | 27,326,009 (90%) | 89.49% | 89.42% |
| C-39 | 34,346,387 (92%) | 33,917,671 (91%) | 32,837,444 (88%) | 92.89% | 92.76% |
| HD-01 | 34,749,722 (94%) | 34,414,796 (93%) | 33,446,726 (90%) | 92.22% | 92.11% |
| HD-02 | 32,703,265 (93%) | 32,244,058 (91%) | 31,557,055 (89%) | 92.33% | 92.25% |
| HD-03 | 36,766,361 (96%) | 36,191,643 (94%) | 35,318,161 (91%) | 92.75% | 92.68% |
| HD-05 | 39,692,486 (97%) | 39,091,303 (95%) | 38,330,890 (93%) | 91.12% | 91.07% |
| HD-06 | 36,273,053 (95%) | 35,654,445 (93%) | 34,919,247 (91%) | 91.59% | 91.53% |
| HD-07 | 27,664,107 (88%) | 27,281,299 (87%) | 26,713,856 (84%) | 88.97% | 88.87% |
| HD-08 | 34,256,457 (97%) | 33,845,324 (96%) | 33,166,289 (93%) | 91.37% | 91.29% |
| HD-09 | 30,795,331 (94%) | 30,390,892 (92%) | 29,826,875 (90%) | 90.20% | 90.11% |
| HD-10 | 30,998,016 (95%) | 30,636,397 (94%) | 30,033,115 (92%) | 89.58% | 89.50% |
| HD-12 | 38,255,463 (93%) | 37,600,000 (91%) | 36,840,523 (89%) | 92.70% | 92.60% |
| HD-13 | 33,597,111 (95%) | 33,166,036 (94%) | 32,174,510 (90%) | 89.63% | 89.50% |
| HD-14 | 58,419,411 (82%) | 57,380,622 (81%) | 55,146,708 (77%) | 90.35% | 90.21% |
| Mean | 34,136,943 (93%) | 33,647,153 (92%) | 32,833,995 (89%) | 90.83% | 90.73% |
